# A Cross‐Sectional Study of Unstable Housing and Housing‐Related Symptom Content in People With Psychosis Admitted for Inpatient Treatment: A Clinical Record Interactive Search Study

**DOI:** 10.1002/hsr2.70189

**Published:** 2024-11-06

**Authors:** Ashley‐Louise Teale, Lucy Docherty, Yasmeen Kenji, Amy Lovell, Pamela Jacobsen

**Affiliations:** ^1^ Taunton and Somerset Foundation Trust Taunton UK; ^2^ Oxford Health NHS Foundation Trust Oxford UK; ^3^ Department of Psychology University of Bath Bath UK

**Keywords:** hallucinations, homeless, paranoia, psychosis, schizophrenia, unstable housing

## Abstract

**Background and Aims:**

Rates of psychosis in the homeless population are markedly higher compared to the general population. Understanding potential psychological mechanisms underpinning links between psychosis and homelessness is important for the development of effective care pathways for this highly marginalised group. This study aimed to examine the housing status of a sample of people with psychosis admitted to psychiatric inpatient hospital in one UK mental health trust. We further aimed to examine the presence and relevance of symptoms which were directly related to housing (e.g., persecutory beliefs about neighbours).

**Methods:**

A cross‐sectional study was conducted using an electronic healthcare database, containing anonymised patient records (Clinical Record Interactive Search). Information on housing status, symptoms, and content of symptoms relating to housing were extracted and independently double‐coded from clinical notes at the person's admission.

**Results:**

351 service users were in the sample, which covered discharges over a 12‐month period (1st April 2021 to 31st March 2022). There was a higher proportion of individuals living without a fixed address in the sample (10%) compared to population estimates (1%–2%). Housing‐related thematic content of symptoms was evident in 34% of the sample (e.g., attributing voices to neighbours, feeling under surveillance at home). The only variable significantly associated with housing status was gender, with men at higher odds of unstable housing compared to women. Individuals with concerns directly related to their housing or housing instability were no more likely to have a longer length of admission than those without housing‐related concerns.

**Conclusion:**

A significant proportion of individuals with psychosis admitted for psychiatric inpatient treatment, experienced housing instability. For some of the sample, symptom content directly related to housing. This raises important considerations for how social needs are assessed on admission to psychiatric hospital. Further research in this area is encouraged.

## 1 Background

Homelessness is a significant societal concern. The term homelessness captures experiences of living without a fixed address, including street sleeping, couch surfing, or staying in temporary accommodations [1]. Rates of psychosis in the homeless population are markedly high, with prevalence estimated at 21.21% [2]. This is much higher than prevalence rates of psychosis in the general population, estimated at 0.9% [3]. This is concerning given mental health disorders and homelessness can contribute to poorer health outcomes [4, 5], including early mortality and suicide [2, 6].

Understanding potential mechanisms underpinning links between psychosis and homelessness is of importance, to present opportunities for prevention and intervention. Experiences of psychosis could result from, contribute to, and maintain housing difficulties. First, research has shown that experiences of disadvantage and experiences of trauma can increase the risk of psychosis onset [7, 8, 9, 10]. The role of cognitive‐affective processes, such as avoidance, hyperarousal and negative other beliefs, have been identified as important in the relationship between trauma and psychosis symptoms [7]. As homelessness is associated with exposure to traumatic events [11, 12], it could be hypothesised that trauma associated with homelessness increases risk of psychosis onset. Although, psychosis may also be a risk factor for unstable housing [13]. Symptoms can be highly distressing and impact upon functioning. This could decrease a person's ability to engage in employment [14], leading to financial difficulties. Additionally, people with psychosis can experience decreased social support and increased isolation [15], which could contribute to lower opportunities for acquiring financial or housing support. The thematic content of positive symptoms (i.e., hallucinations and delusions) could also contribute to housing instability, for example by causing a person to feel unsafe at home. In this context, content might refer to the specific hallucination experienced (e.g., hearing a voice saying the house is unsafe, hearing alarms or visual hallucinations of people in the home) and/or the content of a delusion (e.g., an unfounded belief of being targeted in the home, people breaking in, or the water supply being poisoned). The cognitive model of psychosis suggests that specific symptoms of psychosis, such as persecutory delusions and distressing voices, can trigger safety behaviours, that is, behaviours aimed at maintaining safety, such as avoidance, confrontation and/or other behavioural strategies [16, 17]. These behaviours could make housing untenable, leading to eviction or “voluntary homelessness” where a person leaves with nowhere to go.

Existing evidence has examined risk factors of homelessness. For example, studies have identified the relevance of demographic risk factors, finding being male, socially isolated, of single marital status, older in age and from a minority ethnic background can increase the risk of homelessness [18, 19, 20, 21, 22, 23]. These findings help identify who might be at greatest risk to direct targeted interventions. Existing evidence has also examined risk factors of homelessness among psychosis populations, finding absence of family support and substance use as factors increasing risk [24].

However, to our knowledge there has been limited exploration into specific psychological mechanisms underpinning links between psychosis and housing instability. One study was identified, which examined the role of emotional regulation, maladaptive behaviours (i.e., substance misuse, deliberate self‐harm and high‐risk sexual practices), and the presence/degree of paranoia [25]. There is yet to be research exploring the possible role of the thematic content of symptomology such as hallucinations and delusions, in contributing to homelessness.

Due to possible risks, including suicidality, associated with homelessness and psychosis [2, 6], this population might require admission for psychiatric inpatient treatment. One study found 16% of inpatient admissions experienced homelessness [22]. Evidence suggests homeless individuals use primary care services to a lesser extent than the general population but emergency healthcare at a higher rate [1]. Health inequalities, mistrust and stigma experienced by this group create barriers to accessing health care and seeking help [26, 27, 28]. Therefore, homeless individuals could be less likely to utilise primary mental health care, leading to symptoms increasing in severity, and increasing the need for intensive input. Inpatient services could provide a unique opportunity for accessing and supporting people with housing difficulties, considering this group might not voluntarily present to services.

Housing status is a key consideration in psychiatric care, given housing can play an integral role in recovery. Housing difficulties can also impact the admission, increasing length of stays and instances of delayed discharge, that is, remaining in hospital longer than clinically necessary [29, 30]. Prolonged admissions lead to increased service costs and utilization. In England, the National Health Service (NHS) has emphasised the importance of prioritising timely discharge [31]. As such, examining housing status in inpatient settings, and the impact on length of admission, can inform discharge planning and resource allocation.

Based on the existing evidence base, this study primarily aimed to examine the housing status of people with psychosis admitted to psychiatric inpatient care and the relevance of thematic symptom content in housing instability. We specifically aimed to examine housing‐related content present in positive symptoms of psychosis, that is, concerns relevant to living situation. Positive symptoms included hallucinations, that is, perceptual sensory experiences without external causes and delusions, that is, beliefs held with strong conviction which are considered ‘false.’

We further aimed to examine risk factors of housing difficulties and impact of housing on length of stay, to consider other areas for clinical consideration and intervention. The following research questions were devised:

1. What is the housing status, at the time of psychiatric inpatient admission, of a sample of people with psychosis?
2. What proportion of people with psychosis admitted for psychiatric inpatient treatment, experience positive symptoms which are thematically relevant to their living situation?
3. Is the experience of symptoms of psychosis and presence of housing‐related content in these symptoms associated with housing status, at the time of admission to acute psychiatric inpatient services?
4. Are any demographic variables predictors for housing instability?
5. Does housing status or housing‐relevant symptom content impact length of inpatient admissions?

## 2 Method

### 2.1 Study Design

A retrospective cross‐sectional design was utilised. Data was obtained using Clinical Records Interactive Search (CRIS). This is an established system which enables secure access to in‐depth, routinely collected, and anonymised clinical data [32].

### 2.2 People with Lived Experience Involvement

This project was developed in collaboration with people with experience of living without a fixed address and/or psychosis. This group helped identify relevant variables to examine and emphasised the importance of maintaining anonymity. As such, steps were taken to review data and ensure the write up did not include identifiable experiences.

### 2.3 Sample

For inclusion, individuals needed to be aged 18 or over, admitted to an adult psychiatric inpatient ward, with a diagnosis schizophrenia spectrum disorder on and/or during admission, determined by an ICD‐10 code of F20 to F29. Data was obtained from 11 adult acute, rehabilitation, Psychiatric Intensive Care Units (PICU), and older adult psychiatric wards in Oxford Health NHS Foundation Trust. Data from samples admitted to specialist Learning Disability or forensic inpatient settings was not obtained. An admission summary note with housing status recorded needed to be available for inclusion. Patients' data was extracted if they were discharged during the year audit period (1st April 2021 and 31st March 2022).

### 2.4 Procedure

The study was pre‐registered on the Open Science Framework on 20th October 2022. Data was obtained in November 2022. Data was extracted, cleaned, and organised by three members of the research team (AT, LD, and YK).

First, a data plan was developed with relevant variables to be extracted agreed. For each variable, it was decided whether to extract information from structured in‐put fields or free‐text clinical notes.

To obtain relevant clinical and housing data, at the time of admission, one qualitative free‐text note, the admission summary, was reviewed for each person. This contains information on admission reason, psychiatric presentation (e.g., diagnosis, symptoms, mood), social context (living situation, close contacts), and risk information. Admission summaries might also include relevant historical information, though this was not obtained for this study. It is best practice for the ward's psychiatrist or psychiatric nurse, to complete admission summaries on the day of admission. In this study, if there was not an admission summary within the first 7 days of the admission, another relevant summary note from this timeframe was utilised. If there was no relevant summary in the first 7 days of admission, the person was removed from analysis.

Once located, the clinical note was read in full, with relevant data extracted in accordance with the data plan (see Supporting Information S1: Supplementary Materials). Data was first extracted qualitatively as written on the clinician entered note. Then researchers coded data into pre‐defined categories agreed before extraction. Housing status at admission was categorised into (1) living in fixed accommodation, including sub‐categories of living in rented/owned/school accommodation, living with family, living in supported accommodation and (2) living without a fixed address, encompassing subcategories of street sleeping, staying in temporary accommodation, couch surfing and other, for example, released from/in prison and at risk of eviction. Street sleeping and staying in temporary accommodation were categorised together, as often individuals were utilising both sleeping options interchangeably. Individuals living in inadequate accommodation were coded as living with a fixed address, unless they were at risk of eviction.

Presence of positive symptoms was extracted and organised into the following categories: (1) hallucinations, (2) delusions, (3) both, and (4) no evidence of hallucination/delusion. Binary codes (yes/no) were allocated to whether there was evidence of housing‐related symptom content and housing‐relevant behaviour at admission, that is, safety behaviours related to living situation (e.g., avoiding or altering the home) or behaviours which could risk eviction (e.g., property damage).

### 2.5 Data Validation Process

5% of the sample were randomly selected as training cases. Data from these cases was extracted and coded independently by those tasked with data extraction and coding and reviewed to achieve consistency. Discrepancies were discussed.

Once standardisation was achieved, data extraction and coding were independently conducted by two of the research team and then compared. All the data were rated independently, with discrepancies discussed to reach agreement.

### 2.6 Analysis Method

Data was analysed using R statistical software (RStudio version 1.3.1093).

#### 2.6.1 Research Question One and Two

These research questions were pertaining to prevalence data and were analysed by calculating totals and percentages.

#### 2.6.2 Research Question Three

The prevalence of delusions, hallucinations and housing‐relevant content was calculated with totals and percentages. A Fisher's exact test was utilised to examine associations between positive symptom type and housing stability. A chi‐square test was not appropriate, as the expected frequency within one category was less than five. A chi‐square test of independence was conducted to explore associations between housing‐related content and housing status. Post‐hoc analysis using chi‐square testing was conducted to examine associations between housing‐relevant behaviour and housing status, in addition to housing‐relevant behaviour and housing‐related symptom content.

#### 2.6.3 Research Question Four

A logistic regression was utilised to examine possible risk factors for unstable housing. Based on existing evidence examining risk factors for homelessness, predictor variables examined included gender, ethnicity, marital status, age, and language spoken. To build an explanatory model, a backwards stepwise approach was taken, whereby nonsignificant predictors were removed one‐by‐one until only significant predictors remained [33, 34].

#### 2.6.4 Research Question Five

Independent *t*‐tests were conducted to examine significant differences in length of stay between the following independent variables; (1) housing status (organised into living without a fixed address and living with a fixed address) and (2) housing‐related symptom content (organised into housing‐related content and no housing‐related content). Extreme data scores were not removed from analysis due to the important variability in real‐world clinical data. All tests conducted were two‐tailed with significance value set to *p* < 0.05.

### 2.7 Ethical Approval

NHS trusts utilising CRIS have ethical approval provided from the Health Research Authority. Individual projects are reviewed under each trusts internal committee. This study obtained approval from Oxford Health NHS Foundation Trust research and development department on 11th October 2022.

## 3 Results

Data from 372 individuals was extracted. Twenty‐one individuals were removed as there was no appropriate admission note identified in the timeframe. The final sample consisted of 351 individuals. Sample characteristics are shown (Table 1). Some categories have been combined due to low cell count, to protect anonymity.

**Table 1:** Sample characteristics.

Sample Characteristics	Fixed address *n* (%)	No fixed address *n* (%)	Total *n* (%)
	**315**	**36**	**351**
Male	188 (59.68%)	30 (83.33%)	218 (62.11%)
Female	127 (40.31%)	6 (16.67%)	133 (37.89%)
**Ethnicity**			
White British	160 (50.79%)	17 (47.22%)	177 (50.43%)
Minority ethnic background	100 (31.75%)	9 (25.00%)	109 (31.05%)
Ethnicity not known/recorded	55 (17.46%)	10 (27.78%)	65 (18.52%)
**Primary Language spoken**			
English	104 (29.63%)	7 (19.44%)	111 (31.62%)
Other	16 (4.56%)	4 (11.11%)	20 (5.70%)
Not recorded	195 (55.56%)	25 (69.44%)	220 (62.68%)
**Marital status**			
Single	160 (50.79%)	13 (36.11%)	173 (49.29%)
In partnership/married/not known	155 (49.21%)	23 (63.89)	178 (50.71%)
**Type of setting**			
Acute	240 (76.19%)	28 (77.78%)	268 (76.35%)
Other (i.e., Older Adult, PICU, Rehab)	75 (23.80%)	8 (22.22%)	83 (23.65%)
	**Mean (SD)**	**Mean (SD)**	**Mean (SD)**
Age (in years)	43.58 (15.77)	43.03 (14.88)	43.53 (15.66)
Length of stay (in days)	65.65 (82.78)	68.89 (67.25)	65.98 (81.25)

Abbreviations: PICU, psychiatric intensive care unit.

The sample was predominantly male (62.11%), White British (50.43%), with a mean age of 43.53 (SD = 15.66, Range = 18–88). Most admissions were to general acute psychiatric wards (76.35%) and mean inpatient treatment days were 65.98 (median = 42, SD = 81.25, Range = 1–781).

**Research Q1: What is the housing status, at the time of psychiatric inpatient admission, of a sample of people with psychosis?**

Table two shows the sample's housing status at admission. 315 individuals (89.74%) were living with a fixed address, however, seven of these were living in accommodation inadequate and/or unsafe (Table 2). 36 people (10.26%) were living without a fixed address.

**Table 2:** Housing status of the sample.

Housing Status	*n* (%)
**Living with a fixed address**	**315 (89.74%)**
Rented/owned/school accommodation	144 (41.03%)
Living with family	140 (39.89%)
Supported housing/care home	31 (8.83%)
**Living without a fixed address**	**36 (10.26%)**
Street sleeping/staying in temporary accommodation	25 (7.12%)
Couch surfing	7 (1.99%)
Other (e.g., prison or risk of eviction)	4 (1.14%)

**Research Q2: What proportion of people with psychosis admitted for psychiatric inpatient treatment, experience positive symptoms thematically relevant to their living situation?**

120 people (34.19%) were identified as experiencing housing‐related positive symptom content (Table 3). Examples included:

“believes… bugged his house and installed cameras”

“believes others have tampered with her property… and put gas in the property”

**Table 3:** Prevalence of symptom content thematically relevant to housing, that is, “housing‐related content,” housing‐relevant behaviour and prevalence of positive symptoms.

	Fixed address *n* (%)	No fixed address *n* (%)	Total *n* (%)	Chi‐Square *p value*
	**315**	**36**	**351**	
**Housing‐related content**				
Yes	109 (34.60%)	11 (30.56%)	**120 (34.19%)**	0.76
No	206 (65.40%)	25 (69.45%)	**231 (65.81%)**	
**Housing‐relevant behaviour**				
Yes	84 (26.67%)	12 (33.33%)	**96 (27.35%)**	0.51
No	231 (73.33%)	24 (66.67%)	**255 (72.65%)**	
**Evidence of positive symptom (by type)**				
Delusion	129 (40.95%)	19 (52.78%)	**148 (42.17%)**	0.51
Hallucination	24 (7.62%)	2 (5.56%)	**26 (7.41%)**	
Both	113 (35.87%)	9 (25%)	**122 (34.76%)**	
Delusion/Hallucination not reported	49 (15.56%)	6 (16.67%)	**55 (15.67%)**	

96 individuals (27.35%) were recorded as displaying housing‐related behaviours (Table 3). Examples included:

“Tries to appear more dangerous than he actually is to his neighbours”

“Changed all locks as she thought someone might try to harm her”

“Left home to sleep in a hotel, complaining home felt unsafe”

**Research Q3: Is the experience of symptoms of psychosis and presence of housing‐related content in these symptoms associated with housing status, at the time of admission to acute psychiatric inpatient services?**

Table three shows the presence of hallucinations and delusions in the sample.

Fisher's exact test showed a lack of evidence of an association between positive symptom type and housing status (*p* = 0.51). A chi‐square test of independence did not find a significant relation between housing‐related content and housing status, *χ* ^2^ (1, *N* = 351) = 0.09, *p* = 0.76.

In post‐hoc analysis no significant association was found between housing‐relevant behaviour and housing status: *χ* ^2^ (1, *N* = 351) = 0.43, *p* = 0.51 and no significant association was found between housing‐relevant behaviour and housing‐related symptom content: *χ* ^2^ (1, *N* = 351) = 38.81, *p* = 0.47.

**Research Q4: Are any demographic variables predictors for housing instability?**

In the full model with all predictors present, only gender and language spoken were significant predictors (Table 4). In building the model, variables were removed in the following order: ethnicity, marital status, and age.

**Table 4:** Full logistic regression model.

	Model 1			Final model		
Predictor variable	*p*	OR	95% CI for OR	*p*	OR	95% CI for OR
Gender–female	0.01*	0.28	( > 0.00, 0.25)	0.01*	0.28	(0.11, 0.69)
Ethnicity–minority	0.48	0.70	(0.10, 0.68)			
Ethnicity–not recorded	0.87	0.92	(0.25, 1.82)			
Language–not recorded	0.46	1.45	(0.34, 2.46)			
Language–other	0.03*	5.52	(1.14, 25.55)			
Age	0.25	1.02	(0.99, 1.04)			
Marital status–not known	0.16	3.20	(0.75, 22.26)			
Marital status–single	0.78	1.26	(0.31, 8.53)			

When only primary language spoken and gender remained, language was no longer significant and was removed from the explanatory model. The only predictor variable remained was gender (*p* < 0.05). There was a significant difference between males and females in housing stability, when adjusting for other variables.

**Research Q5: Does housing status or housing‐relevant symptom content, impact length of inpatient admissions?**

There was no significant difference in length of stay between those with or without a fixed address (mean length of stay of 66 days for those with a fixed address vs. 69 days for those without a fixed address, *p* = 0.79).

There was no significant difference in length of stay between groups with or without housing‐related content (mean length of stay of 65 days for those with housing‐related content vs. 66 days for those without, *p* = 0.92).

See Table 5 for additional data.

**Table 5:** Length of admission (in days) by housing status and housing‐related symptom content.

	Mean (SD)	Median	Min	Max	*t* (df)	*p Value*
**Total sample**	**65.98 (81.24)**	**42**	**1**	**781**		
**Housing status**						
Fixed adress	65.65 (82.78)	41	1	781	−0.27 (48.01)	0.79
Not fixed address	68.89 (67.52)	55	1	343		
**Housing‐related content**						
Yes	65.43 (67.59)	41.0	1	397	0.10 (299.46)	0.92
No	66.27 (87.65)	42.5	1	781		

## 4 Discussion

This study aimed to examine housing status of people with psychosis admitted for psychiatric inpatient treatment, and presence of housing‐related content in positive symptomology. Further aims were to examine relevance of housing‐related content in housing instability, risk factors of homelessness and the impact of housing instability on length of stay. This study hoped to add to evidence examining links between psychosis and homelessness.

10.26% of the sample were living without a fixed address at admission. This is much higher than rates of homelessness in the general population, estimated to be under 2% [35]. The findings suggest increased vulnerability of individuals with psychosis to experience difficulties securing and maintaining housing. However, rates of homelessness were lower compared to previous work identifying homelessness in 16% of acute psychiatric admissions in South London [22]. This lower rate could indicate a reduction in homelessness since 2012 or be representative of the locality examined, as London experiences the highest homelessness rates in the UK [36]. The lower rate could also be because the present study examined psychosis admissions specifically not all psychiatric admissions, unlike Tulloch and colleague's paper, therefore the difference could be due to sampling differences.

34.19% of the sample were identified as experiencing housing‐related concerns and 27.35% housing‐related behaviours. Therefore, over a quarter had beliefs/hallucinations related to their living situation and over a quarter displayed behaviours related to living situation. However, no statistically significant association was found between housing status and these variables, providing no evidence these psychological mechanisms underpin links between psychosis and homelessness. This is surprising, as it might be expected those who experience thematic symptom content related to housing and/or housing‐relevant behaviour, would be at greater risk of housing instability. It might be the case that housing‐related content and behaviour was less relevant to those currently living without a fixed address or not assessed/recorded at admission in the same way. For example, concerns about neighbours, intruders, could be less relevant to the current living situation of having no fixed address and historical beliefs were not captured in this study. It could also be that other relevant factors might be of greater importance, such as specific content of housing‐related symptoms, the type of symptom (i.e., persecutory belief, auditory hallucination) or safety behaviour (i.e., confrontation, avoidance), or severity of distress. Future research could collect more nuanced data with further categorisation, beyond binary categories. Previous work examined the role of emotional regulation and maladaptive behaviours, i.e., substance misuse, deliberate self‐harm and high‐risk sexual practices [25]. In our sample, we did not explore these variables. These factors could have underpinned links in our sample. Furthermore, methodological considerations described below, might have contributed towards these results.

In this study, males were at significantly greater risk of living without a fixed address compared to females. This links to existing evidence which highlighted only 13% of people street sleeping in England are female [37]. As this is comparable to our findings, there could be some indication males are not disproportionately disadvantaged due to psychosis. However, no other demographic variables (marital status, ethnicity, primary language spoken) were significant predictors of housing instability. There was also no association between housing stability and length of stay. This does not support existing evidence. It is plausible the number of people in the sample experiencing insecure housing was not sufficient to establish an equitable basis for comparison with those securely housed, to identify relevant risk factors.

This study offers some important psychosocial considerations for psychiatric inpatient treatment. The prevalence of housing instability emphasises the need for services to assess housing status and consider possible avenues of support at admission. Inpatient settings could provide a unique opportunity for intervention, particularly given those experiencing homelessness might be less likely to present to primary care services [1]. The Royal College of Psychiatrists (2017) Standards for Inpatient Care state acute psychiatric care services should support inpatients with housing. As such, inpatient settings could offer support to prevent loss of housing and identify appropriate accommodation. Ensuring clear policy, standard operating procedures, and guidance for how to support homeless individuals in inpatient services would be of benefit, particularly because inpatient care could provide a unique opportunity for intervention and support. Clearer guidance would better enable services to offer appropriate support and consider the best care pathways. In addition, the finding that over one quarter of the sample experienced housing‐related content and housing‐relevant behaviour presents a novel finding, emphasising the importance of assessing symptom content and behavioural responses at admission. It also suggests that individual formulation and intervention, for example with safety behaviours, could be of clinical importance.

When interpreting findings, there are key considerations to note. Clinicians might not have explicitly assessed or recorded housing related concerns, behaviours, or symptom content at admission. Addressing immediate psychiatric symptoms can take precedence over examining ancillary concerns [38], such as housing stability. As there is not a standardised process regarding what to record in clinical notes, it is unclear to what extent this might impact the findings. Individuals also might not have proactively shared housing‐related difficulties or concerns during the admission process. Another key consideration is the measurement of variables at the time of admission. This offered a fixed time to record housing status and clinical presentation, though does not offer a historical or longitudinal account of experiences. It should also be noted there are considerable barriers faced by people with housing difficulties in accessing mental health support, for example, stigma and logistical barriers. This study did not capture people who did not access services, or reach crisis point to require admission. The findings therefore might not extend to these groups. Finally, there is limited generalisability given the limited sample size, and given this study covered one single health‐care region in England.

Strengths of the study include using a broad definition of the term “homelessness” to recognise the varying living situations experienced. Methodological strengths include utilising CRIS to support understanding of real‐life experiences and completing inter‐rating for the entirety of the data set.

More research on examining relevant psychological mechanisms which might underpin links between psychosis and homelessness is indicated, to enhance understanding, given there has been little focus to date. Such understanding would support recommendations for prevention and intervention. Future research could explore possible psychological mechanisms via qualitative interviews or questionnaires, to direct quantitative research and identify mechanisms for further exploration. Exploring housing difficulties for individuals with psychosis in community services and those not accessing services would be of benefit to consider the population not captured in this study. There might be differences across groups, as people accessing services might have increased opportunities for housing support, which could maintain housing. Finally, it would be of benefit for longitudinal evaluation, as this study only examined one time point. Some people in this study might have experienced homelessness previously or went on to experience homelessness after admission, so longitudinal examination might yield different findings.

In conclusion, 10% of the sample were living without a fixed address and 34% presented with delusions/hallucinations where thematic content related to housing (i.e., housing‐related content). This highlights how over a quarter of people admitted for treatment, regardless of housing stability, experience housing‐related concerns. This is important for clinical assessment and intervention. However, there was no association found between housing‐related content and housing status. Therefore, this study did not find evidence to support that thematic content of symptoms (hallucinations and delusions) underpins links between psychosis and homelessness. Future research and continued focus in this area is encouraged to develop best care practices for people with psychosis experiencing housing difficulties.

## Author Contributions

Ashley‐Louise Teale and Pamela Jacobsen developed the initial research questions, with support from an expert by experience (Amy Lovell). Ashley‐Louise Teale, Pamela Jacobsen, and Amy Lovell developed the study protocol. Ashley‐Louise Teale, Lucy Docherty, and Yasmeen Kenji completed data cleaning, coding and analysis. Ashley‐Louise Teale, Lucy Docherty, Yasmeen Kenji and Pamela Jacobsen contributed to data interpretation. Ashley‐Louise Teale wrote the paper. All authors read and approved the final version of manuscript for submission. Dr. Ashley‐Louise Teale (corresponding/lead author) had full access to all of the data in this study and takes complete responsibility for the integrity of the data and the accuracy of the data analysis.

## Conflicts of Interest

The authors have no competing interests to declare.

## Transparency Statement

The lead author Ashley‐Louise Teale affirms that this manuscript is an honest, accurate, and transparent account of the study being reported; that no important aspects of the study have been omitted; and that any discrepancies from the study as planned (and, if relevant, registered) have been explained.

## Supporting information

Supporting information.

## Data Availability

This study was supported by CRIS Powered by Akrivia Health, using data, systems and support from the NIHR Oxford Health Biomedical Research Centre (NIHR203316) Research Informatics Team. The source data for this work is owned by Oxford Health NHS Foundation Trust using anonymised patient records via CRIS Powered by Akrivia Health. The data cannot be made publicly available but can be accessed with permissions from Oxford Health NHS Foundation Trust for UK NHS staff and UK academics within a secure firewall, in the same manner as the authors.
