# Supplementary material for: A Cross‐Sectional Study of Unstable Housing and Housing‐Related Symptom Content in People With Psychosis Admitted for Inpatient Treatment: A Clinical Record Interactive Search Study
Source: Health Sci Rep. 2024 Nov 6;7(11):e70189. doi: 10.1002/hsr2.70189 (PMC11540838; doi:10.1002/hsr2.70189)
Supplement: Supplementary file 1 — Supporting information. [file HSR2-7-e70189-s001.docx]

| **Supplementary Materials**  Data Extraction Plan | |  |  |
| --- | --- | --- | --- |
| **Variable Extracted** | **Definition of the variable extracted** | **Extraction location** | **Categories** |
| **Gender** | Self-reported gender of the individual | Structured input field | Female  Male  Non-Binary  Transgender  Other |
| **Ethnicity** | Self-reported ethnicity of the individual | Structured input field | Asian or Asian British  Black or Black British  Mixed  Not known  Other ethnic groups  White - Other  White – British |
| **Primary Language Spoken** | Self-reported primary language spoken of the individual | Structured input field | English  Other  Not Recorded |
| **Sexual Orientation** | Self-reported sexual orientation of the individual | Structured input field | Bi-sexual  Gay/Lesbian  Heterosexual  Not stated  Other sexual orientation not listed |
| **Marital status** | Self-reported marital status of the individual | Structured input field | Single  In partnership/married  Not known |
| **Date of birth** | Date of birth | Structured input field | - |
| **Age** | Age at admission | Calculated via date of birth and date of admission | - |
| **Psychosis code** | Diagnostic code allocated by psychiatrist/clinical team on or during admission | Structured input field | F20 = Schizophrenia  F21 = Schizotypal F22 = Delusional disorder F23 = Acute and transient psychotic disorder F25 = Schizoaffective disorder  F28 = Other non-organic psychosis F29 = Unspecified psychosis |
| **Date of Admission** | Date patient admitted to inpatient service | Structured input field | - |
| **Date of discharge** | Date patient discharged | Structured input field | - |
| **Days admitted** | Number of days patient admitted to service | Calculated via date of discharge and date of admission | - |
| **Type of inpatient setting** | Type of adult inpatient setting the person was admitted too. | Structured input fields | Older adult  Adult acute  Rehabilitation  PICU  Mixed – i.e., combination of different settings via transfer |
| **Type of Note** | Type of clinical note utilised for free text data extraction. | Categorised by researcher | Admission Clerking  Admission Assessment  Admission Summary  Consultant Review  Psychiatric Liaison Service  Ward Round |
| **Date of clinical note** | Date of the clinical note utilised for free text data extraction. | - | - |
| **Housing status** | Qualitative description of the person’s housing status, e.g., living with parents, living in rented accommodation, staying in temporary accommodation | Clinical note – Admission summary or other relevant note within the first seven days of the admission | Qualitative data |
| **Housing status: Binary Code** | Categorisation of the person’s housing status by research team based on the qualitative description in clinical notes. | Clinical note – Admission summary or other relevant note within the first seven days of the admission  Then categorised by research team | Fixed address, including – living in rented/owned accommodation; living with family; school/university accommodation; supported housing/care home  Non-fixed address – street-sleeping/staying in temporary accommodation, couch surfing, prison, at risk of eviction |
| **Evidence of hallucinations** | Is there evidence of: Hallucinations, i.e., an perceptual sensory experience in the absence of an external cause | Clinical note – Admission summary or other relevant note within the first seven days of the admission | Yes  No |
| **Hallucinations: Auditory** | Is there evidence of: Hearing voices or sounds that others do not hear? | Clinical note – Admission summary or other relevant note within the first seven days of the admission | Yes  No |
| **Hallucinations: Visual** | Is there evidence of: Perceiving objects, people, or events that are not present in reality? | Clinical note – Admission summary or other relevant note within the first seven days of the admission | Yes  No |
| **Hallucinations: Olfactory** | Is there evidence of: Sensing smells or odours that are not present? | Clinical note – Admission summary or other relevant note within the first seven days of the admission | Yes  No |
| **Hallucinations: Gustatory** | Is there evidence of: Perceiving tastes that are not present? | Clinical note – Admission summary or other relevant note within the first seven days of the admission | Yes  No |
| **Hallucinations: Tactile** | Is there evidence of: Feeling sensations on the skin without any external cause? | Clinical note – Admission summary or other relevant note within the first seven days of the admission | Yes  No |
| **Evidence of delusions?** | Is there evidence of: Delusions i.e., a belief held with strong conviction in the absence of evidence or is considered ‘false.’ | Clinical note – Admission summary or other relevant note within the first seven days of the admission | Yes  No |
| **Delusions: Persecutory** | Is there evidence of: Believing that one is being targeted, watched, or conspired against? | Clinical note – Admission summary or other relevant note within the first seven days of the admission | Yes  No |
| **Delusions: Grandiose** | Is there evidence of: Having exaggerated beliefs of power, importance, or special abilities? | Clinical note – Admission summary or other relevant note within the first seven days of the admission | Yes  No |
| **Delusions: Somatic** | Is there evidence of: Believing in false physical health conditions or bodily changes? | Clinical note – Admission summary or other relevant note within the first seven days of the admission | Yes  No |
| **Delusions: Control** | Is there evidence of: Believing that one's thoughts, feelings, or actions are controlled by external forces? | Clinical note – Admission summary or other relevant note within the first seven days of the admission | Yes  No |
| **Delusions: Monitoring** | Is there evidence of: Believing in being monitored/followed? | Clinical note – Admission summary or other relevant note within the first seven days of the admission | Yes  No |
| **Experience of hallucination or delusion** | Is there evidence of person experiencing a hallucination or delusion at admission? | Clinical note – Admission summary or other relevant note within the first seven days of the admission | Hallucination  Delusion  Both  Neither |
| **Other?** | Is there evidence of other symptoms, e.g., risk, negative symptoms, catatonia, mania, etc? | Clinical note – Admission summary or other relevant note within the first seven days of the admission | Qualitative data |
| **Housing-Related Concerns** | Clinical note indicates patient’s expressing hallucinations/delusions whereby the content thematically links to housing, e.g. surveillance by neighbours, sabotage of living conditions, intruders. | Clinical note – Admission summary or other relevant note within the first seven days of the admission | Qualitative description recorded and then coded to: Evidence of housing related concern:  Yes  No |
| **Housing-Related Behaviour** | Clinical note indicates patient engaging in behaviour at their home which could be risky/impact on housing stability, e.g. vandalizing or damaging one's own home or property, neglecting maintenance of the house (leading to unsafe living conditions), hoarding or excessive clutter, or engaging in safety behaviour that involves altering the home. | Clinical note – Admission summary or other relevant note within the first seven days of the admission | Qualitative description recorded and then coded to: Evidence of housing related behaviour:  Yes  No |
